# Circulating Microbiota in Cardiometabolic Disease

**DOI:** 10.3389/fcimb.2022.892232

**Published:** 2022-05-03

**Authors:** Keiichi Sumida, Zhongji Han, Chi-Yang Chiu, Tahliyah S. Mims, Amandeep Bajwa, Ryan T. Demmer, Susmita Datta, Csaba P. Kovesdy, Joseph F. Pierre

**Affiliations:** ^1^ Division of Nephrology, Department of Medicine, University of Tennessee Health Science Center, Memphis, TN, United States; ^2^ Department of Preventive Medicine, University of Tennessee Health Science Center, Memphis, TN, United States; ^3^ Department of Nutritional Sciences, College of Agriculture and Life Science, University of Wisconsin-Madison, Madison, WI, United States; ^4^ Transplant Research Institute, James D. Eason Transplant Institute, Department of Surgery, School of Medicine, University of Tennessee Health Science Center, Memphis, TN, United States; ^5^ Division of Epidemiology and Community Health, School of Public Health, University of Minnesota, Minneapolis, MN, United States; ^6^ Department of Epidemiology, Mailman School of Public Health, Columbia University, New York, NY, United States; ^7^ Department of Biostatistics, University of Florida, Gainesville, FL, United States; ^8^ Nephrology Section, Memphis Veterans Affairs (VA) Medical Center, Memphis, TN, United States

**Keywords:** cardiometabolic disease, cardiovascular disease, chronic kidney disease, circulating microbiota, diabetes mellitus, end-stage kidney disease, gut microbiota, inflammation

## Abstract

The rapid expansion of microbiota research has significantly advanced our understanding of the complex interactions between gut microbiota and cardiovascular, metabolic, and renal system regulation. Low-grade chronic inflammation has long been implicated as one of the key mechanisms underlying cardiometabolic disease risk and progression, even before the insights provided by gut microbiota research in the past decade. Microbial translocation into the bloodstream can occur *via* different routes, including through the oral and/or intestinal mucosa, and may contribute to chronic inflammation in cardiometabolic disease. Among several gut-derived products identifiable in the systemic circulation, bacterial endotoxins and metabolites have been extensively studied, however recent advances in microbial DNA sequencing have further allowed us to identify highly diverse communities of microorganisms in the bloodstream from an -omics standpoint, which is termed “circulating microbiota.” While detecting microorganisms in the bloodstream was historically considered as an indication of infection, evidence on the circulating microbiota is continually accumulating in various patient populations without clinical signs of infection and even in otherwise healthy individuals. Moreover, both quantitative and compositional alterations of the circulating microbiota have recently been implicated in the pathogenesis of chronic inflammatory conditions, potentially through their immunostimulatory, atherogenic, and cardiotoxic properties. In this mini review, we aim to provide recent evidence on the characteristics and roles of circulating microbiota in several cardiometabolic diseases, such as type 2 diabetes, cardiovascular disease, and chronic kidney disease, with highlights of our emerging findings on circulating microbiota in patients with end-stage kidney disease undergoing hemodialysis.

## Introduction

Cardiometabolic disease, including type 2 diabetes, chronic kidney disease (CKD), and cardiovascular disease is a significant global healthcare problem with growing prevalence and substantial social and economic burden (Aron-Wisnewsky and Clément, 2016; Ralston and Nugent, 2019). The number of people with cardiovascular disease, type 2 diabetes, and CKD worldwide is reported to be 523 million (Roth et al., 2020), 422 million (Collaboration, 2016), and 847 million (Jager et al., 2019), respectively. In 2010, the global financial burden of cardiometabolic disease was estimated to be US$6.3 trillion, which is projected to double by 2030 (Arena et al., 2015).

Cardiometabolic disease stems from various factors, including genetic, behavioral and environmental factors (Ralston and Nugent, 2019). Low-grade chronic inflammation represents a key pathophysiological mechanism shared in common between the various cardiometabolic disease entities (Donath et al., 2019). A variety of mechanisms have been suggested to contribute to the perpetuation of inflammatory responses in cardiometabolic disease, including release of adipokines from obese visceral adipose tissue, renin-angiotensin-aldosterone system (RAAS) activation, cellular senescence, and accumulation of toxic metabolites (Carrero and Stenvinkel, 2010; Oishi and Manabe, 2020; Sumida et al., 2020). Over the past few decades, substantial efforts have been made to alleviate the chronic inflammation in cardiometabolic disease mainly by targeting these etiological factors, which have not been very successful; and the considerable disease burden resulting from chronic inflammation remains to be resolved. Therefore, an urgent need exists to identify novel modifiable risk factors that could help develop effective therapeutic approaches for premature morbidity and mortality in patients with cardiometabolic disease.

With recent advances in ‘-omics’ technologies, bioinformatics, and modelling approaches, a growing body of evidence suggests that microbial communities (i.e., microbiota) along the digestive tract may contribute to chronic low-grade inflammation in cardiometabolic disease (Aron-Wisnewsky and Clément, 2016; Warmbrunn et al., 2020), which in turn suggests that the microbiota could serve as a novel therapeutic target against cardiometabolic disease (Fernández-Ruiz, 2021; Sumida et al., 2021a). In a recent prospective study examining the long-term effects of a Mediterranean-style diet on the gut microbiome composition and on cardiometabolic disease risk (i.e., glucose homeostasis, lipid metabolism and inflammation), a healthy Mediterranean-style dietary pattern modified the risk of cardiometabolic disease in part through alterations of the gut microbiota (Wang et al., 2021). Given the enormous microbial load (i.e., >100 trillion individual microorganisms) in the human gastrointestinal tract and their substantial modulation of most metabolic activities (Whitman et al., 1998), it is not surprising that many diseases, including cardiometabolic disease, are related to altered gut microbiota (a.k.a. gut dysbiosis) and resultant changes in gut-derived metabolites. In contrast, the contributions of extraintestinal microbial communities circulating in the blood, which is also known as “circulating microbiota”, have been scarcely documented, let alone explored for their potential pathophysiological role in cardiometabolic disease. However, evidence that supports the roles of circulating microbiota in the onset and progression of cardiometabolic disease is steadily accumulating and receiving increasing attention. In this mini review, we summarize the current understanding of the circulating microbiota in shaping the development and progression of cardiometabolic disease with clinical and research implications from this rapidly evolving field, and highlight some of the emerging findings on the association of circulating microbiota with risk of cardiovascular disease in patients with end-stage kidney disease (ESKD) on hemodialysis.

## Circulating Microbiota

While the colonization of microbes at specific body sites that are exposed to the external environment (e.g., the oral cavity and the gut) is both well-recognized and widely accepted (Markova, 2017), the concept of presence of microbial communities in an otherwise “sterile” milieu, such as the bloodstream, is relatively new. Traditionally, the detection of microbes in the bloodstream carried out by culturing specific microbes is interpreted as an indication of infection. However, the concept of the existence of classically “sterile” milieu in the blood of healthy humans has been challenged by mounting evidence showing the existence of blood microbes in otherwise healthy individuals (McLaughlin et al., 2002; Damgaard et al., 2015; Paisse et al., 2016). Following the seminal study by Nikkari et al. in 2001 that reported the detection of bacterial DNA in blood specimens from healthy individuals (Nikkari et al., 2001), several studies have reported the presence of blood microbes among both healthy blood donors (McLaughlin et al., 2002; Damgaard et al., 2015; Paisse et al., 2016) and various patient populations without overt infections (Rajendhran et al., 2013; Sato et al., 2014; Lelouvier et al., 2016), primarily by amplification and sequencing of the bacterial 16S ribosomal RNA (rRNA) gene. The application of archaeal 16S rRNA and fungal Internal Transcribed Spacer (ITS) rRNA sequencing and whole-genome shotgun sequencing techniques have also demonstrated the presence of archaea, fungi, and viruses in blood of healthy individuals (Dinakaran et al., 2014; Panaiotov et al., 2018; Castillo et al., 2019). It is important to note that the detection of microbial signatures in these studies is based largely on microbial DNA signatures and not viable bacteria directly, and hence do not necessarily challenge existing dogma, but rather provide deeper insights into the concept of sterility and homeostasis in the cardiovascular system.

## Sources of Circulating Microbiota

The source of a circulating microbiota remains a topic of considerable deliberation, and it is still controversial whether the circulating microbiota is allochthonous or autochthonous (Castillo et al., 2019). Currently, the presence of circulating microbiota is thought to be largely attributable to microbial translocation from other body sites rich in microbiota (Velmurugan et al., 2020). The initial entry of microorganisms into the systemic circulation has been thought to occur prenatally by vertical transmission of maternal microbiotas in the umbilical cord, placenta, and/or amniotic fluid, although the conventional dogma suggests that the placental barrier prevents such a microbial translocation into infants during pregnancy (Funkhouser and Bordenstein, 2013). The other possible major sources of circulating microbiota include gastrointestinal tract, mouth, and skin (Whittle et al., 2018). Aside from the microbial translocation as a result of injuries, infections, and non-surgical procedures (e.g., intravenous injections and catheter placement), microbial translocation from these body sites is most likely to occur through loosening of epithelial barriers due to their structural and functional alterations. For example, microbial translocation from the oral cavities into the systemic circulation has been reported in oral diseases, such as gingivitis and periodontitis (Emery et al., 2021), and even after chewing, tooth brushing and dental flossing, all of which can lead to the oral mucosal barrier disruption (Forner et al., 2006; Lockhart et al., 2008). The identification of known members of the oral microbiota in the coronary artery tissues from patients with sudden cardiac death may also support these findings (Lehtiniemi et al., 2005), and prior work has also demonstrated the recovery of viable bacteria from atheromatous plaques (Rafferty et al., 2011).

The impairment of intestinal barrier integrity is another important mechanism that allows microbial translocation from the gut into the systemic circulation. This so-called “leaky gut” phenomenon has been well-studied and recognized in certain populations, such as those with inflammatory bowel disease (Maloy and Powrie, 2011), portal hypertension (Lutz et al., 2015), and heart failure (Verbrugge et al., 2013; Yuzefpolskaya et al., 2020). In recent years, this phenomenon has also been recognized in a variety of chronic diseases and conditions, including diabetes mellitus, CKD, and cardiovascular disease, partly due to gut dysbiosis, lifestyle and dietary factors (e.g., high-fat diet and alcohol intake), medications (e.g., non-steroidal anti-inflammatory drugs), and intestinal ischemia and/or edema (de Kort et al., 2011; Kitai and Tang, 2018; Nagpal et al., 2018; Sumida and Kovesdy, 2019). Among possible gut-derived products that can be identified in the bloodstream, bacterial lipopolysaccharide (i.e., endotoxin) and metabolites (e.g., indoxyl sulfate and trimethylamine N-oxide) have been most extensively investigated for their unique atherogenic and immunostimulatory properties (Evenepoel et al., 2009; Wiesner et al., 2010; Tang et al., 2013; Bowman et al., 2017; Donath et al., 2019); however, the translocation of gut microbiota into the systemic circulation may also occur as a result of the increased gut permeability, as supported by a few studies demonstrating a similarity in the microbial signatures between the fecal and blood samples of patients with cardiometabolic disease (Sato et al., 2014; Lelouvier et al., 2016). Importantly, both quantitative and compositional changes in the microbiota translocated into the systemic circulation have been implicated in the pathogenesis of cardiometabolic disease.

## Circulating Microbiota and Cardiometabolic Disease

### Changes in Circulating Microbial DNA Levels and Cardiometabolic Disease

Bacterial DNA fragments can be detected by amplifying the highly conserved 16S rRNA subunit or through whole genome sequencing approaches (Jiang et al., 2009). Among various microbial components, bacterial DNA fragments are readily detectable and are easily discerned from human DNA (Szeto et al., 2018), and have thus been suggested as a better quantitative marker of the bacterial load circulating in the blood compared to measuring bacterial lipopolysaccharide (i.e., endotoxin) which is limited to detect only Gram-negative bacteria (Wiedermann et al., 1999). Furthermore, circulating bacterial DNA has been suggested to have unique pathogenic roles through recognition as pathogen-associated molecular pattern ligands (PAMPs) in host immune and cardiovascular systems.

Bacterial DNA contains unmethylated cytosine–guanine dinucleotide (CpG) flanked by two purine 5’ and two pyrimidine 3’ (Klinman et al., 1997). These DNA structures are recognized by toll-like receptors (TLRs), specifically endogenous TLR-9 (as a bacterial DNA receptor). This in turn triggers a cell signaling pathway including activation of the nuclear factor kappa B and the mitogen-activated protein kinases (Krieg, 2002). In inflammatory immune cells like polymorphonuclear neutrophils, bacterial DNA products exert profound effects on chemokine expression, regulation of adhesion molecules, cellular trafficking, and phagocyte activity. They promote the survival of mononuclear cells by inducing interleukin-6 (IL-6) (Schindler et al., 2004; Navarro et al., 2007) and rescues polymorphonuclear neutrophils from constitutive apoptosis (El Kebir et al., 2008). The activation of host immune system induced by bacterial DNA can in turn induce a plethora of other compounding pathological pathways that include metabolic dysfunction (e.g., through insulin resistance) and endothelial injury (e.g., through induction of endothelial cell apoptosis) (Merino et al., 2008; Donath et al., 2019), eventually contributing to the pathogenesis of cardiometabolic disease. In fact, elevated levels of circulating bacterial DNA have been reported in individuals with cardiometabolic disease who had no clinical evidence of systemic infection (Amar et al., 2011; Kwan et al., 2013; Dinakaran et al., 2014; Szeto et al., 2018).

In addition to its proinflammatory and atherogenic properties, it has been shown that bacterial DNA induces suppression of cardiac myocyte contraction in a dose-dependent manner (Paladugu et al., 2004). In line with this observation, higher plasma bacterial DNA levels have been demonstrated to be independently associated with subsequent risk of composite cardiovascular events (comprised mostly of hospitalization for heart failure) in peritoneal dialysis patients without overt infections (Szeto et al., 2015). Of interest, compared with bacterial endotoxin levels, the association with cardiovascular events was more pronounced for bacterial DNA levels (Szeto et al., 2015), suggesting that circulating bacterial DNA could play a more dominant role in the development and progression of cardiovascular disease.

### Changes in Circulating Microbial Composition and Cardiometabolic Disease

Compositional changes in the microbiota are usually represented by alpha (α) diversity (e.g., assessed by Shannon, Simpson, Chao1 and Richness indices) and relative abundance of microbial communities. While the advent of sequencing technology has greatly advanced our understanding of the roles of compositional alterations of the gut microbiota in various diseases (Ley et al., 2006; Schwabe and Jobin, 2013; Ramezani and Raj, 2014; Wang and Kasper, 2014; Autenrieth, 2017), studies reporting the compositional changes in circulating microbiota and their association with cardiometabolic disease are still extremely limited (**Table 1**).

**Table 1:** Reported compositional changes in circulating microbiota associated with cardiometabolic disease.

Cardiometabolic disease	Associated changes in circulating microbiota	Detection methods	Samples used	Reference
Type 2 diabetes	Proteobacteria phylum represented the highest relative abundance (~90%) *Ralstonia* was the most dominant genus within the Proteobacteria phylum	Amplicon sequencing for V1–V2 regions of bacterial 16S rRNA	Peripheral blood leucocytes	(Amar et al., 2011)
	Proteobacteria phylum represented the highest relative abundance (~99%) *Aquabacterium*, *Pseudonocardia*, and *Xanthomonas* genera were lower, while *Alishewanella*, *Actinotalea*, *Pseudoclavibacter*, and *Sediminibacterium* genera were higher in patients with type 2 diabetes than non-diabetic individuals	Amplicon sequencing for V5–V6 regions of bacterial 16S rRNA	Plasma	(Qiu et al., 2019)
Cardiovascular disease	Higher Proteobacteria phylum was associated with higher risk of incident cardiovascular events	Amplicon sequencing for bacterial 16S rRNA	Peripheral blood leukocytes	(Amar et al., 2013)
	Proteobacteria phylum was higher in patients with cardiovascular disease than healthy individuals	Amplicon sequencing for V3 region of bacterial 16S rRNA	Whole blood	(Rajendhran et al., 2013)
	Norcardiaceae and Aerococcaceae families and *Gordonia*, *Propionibacterium*, *Chryseobacterium*, and *Rhodococcus* genera (cholesterol-degrading bacteria) were lower in patients with (vs. without) myocardial infarction	Amplicon sequencing for V3–V4 regions of bacterial 16S rRNA	Whole blood	(Amar et al., 2019)
Chronic kidney disease	Proteobacteria phylum, Gammaproteobacteria class, and Enterobacteriaceae and Pseudomonadaceae families were higher in patients with CKD than healthy individuals	Amplicon sequencing for V3–V4 regions of bacterial 16S rRNA	Buffy coat	(Shah et al., 2019)
End-stage kidney disease	Proteobacteria and Actinobacteria phyla were lower and higher, respectively, in ESKD patients with (vs. without) a fatal cardiovascular disease	Amplicon sequencing for V3–V4 regions of bacterial 16S rRNA	Serum	(Sumida et al., 2021b)

CKD, chronic kidney disease; ESKD, end-stage kidney disease; rRNA, ribosomal RNA.

#### Circulating Microbiota in Type 2 Diabetes

Reports on the circulating microbiota in diabetes relate exclusively to type 2 diabetes, and to our knowledge no correlation studies have been reported in type 1 or gestational diabetes. In a case-control study nested from a longitudinal cohort study examining the association of blood 16S rDNA concentrations with incident diabetes and obesity in non-diabetic and non-obese adults, the pyrosequencing analysis demonstrated that Proteobacteria phylum represented the highest relative abundance (~90%) in the blood of individuals who developed diabetes, with *Ralstonia* being the most dominant genus within the Proteobacteria phylum (Amar et al., 2011). In a recent nested case-control study of 15 cases with incident type 2 diabetes and 100 matched controls without type 2 diabetes, the researchers investigated the composition of the circulating microbiota using bacterial 16S rRNA sequencing and found that both cases and controls had diverse bacterial communities in their pre-diagnostic blood samples, dominated largely by Proteobacteria phylum (~99%) (Qiu et al., 2019). Although there was no difference in α diversity (assessed by Simpson, Shannon, and Chao1 indices) between the two groups, the relative abundance of *Aquabacterium*, *Pseudonocardia*, and *Xanthomonas* genera was significantly lower among diabetic cases compared with non-diabetic controls, while that of *Alishewanella*, *Actinotalea*, *Pseudoclavibacter, and Sediminibacterium* genera was significantly higher among cases than controls. Of interest, the study also showed that individuals carrying the *Bacteroides* and *Sediminibacterium* genera in the blood at baseline had significantly lower and higher risk of incident type 2 diabetes, respectively, independent of potential confounders (Qiu et al., 2019), although precise mechanisms underlying these associations remain to be determined.

#### Circulating Microbiota in Cardiovascular Disease

The profiles of blood microbes in the context of cardiovascular disease were traditionally restricted to infection-related cardiac complications, such as rheumatic heart disease and infectious peri-, myo- and endocarditis, diagnosed primarily by classical serological and/or culture-based methods (Fong, 2009). However, recent evidence regarding the association between circulating microbiota and cardiovascular disease has questioned this paradigm that links the presence of blood microbes to infectious cardiac complications.

In a pioneering population-based study examining the longitudinal association of circulating microbiota identified using the 16S rRNA sequencing with cardiovascular events in 3,936 adults without clinical evidence of infection, there was a significant association between relative abundance of Proteobacteria phylum (identified in peripheral blood leukocytes) and risk of incident cardiovascular events, independently of traditional cardiovascular risk factors (Amar et al., 2013). Subsequently, a cross-sectional study reported similar findings that, compared with apparently healthy individuals, patients with cardiovascular disease had a significantly higher relative abundance of Proteobacteria phylum in their whole blood (Rajendhran et al., 2013). Although these observational studies cannot conclude a causal relationship, there are several plausible explanations for the association between circulating microbiota and cardiovascular disease. In addition to the aforementioned immunostimulatory, atherogenic, and cardiotoxic properties of bacterial DNA fragments, the phylum Proteobacteria, which was found to be predominant in the blood of patients with cardiovascular disease in previous studies, has unique proinflammatory and proatherosclerotic properties (Rizzatti et al., 2017) and may thus, as a culprit pathogen, contribute to the development and progression of cardiovascular disease. Additionally, a decrease in the relative abundance of cholesterol-degrading bacteria, such as Norcardiaceae and Aerococcaceae families and *Gordonia*, *Propionibacterium*, *Chryseobacterium*, and *Rhodococcus* genera, has been implicated in the pathogenesis of atherosclerotic plaques, leading to the risk of ischemic heart disease (Amar et al., 2019).

#### Circulating Microbiota in Chronic Kidney Disease

Patients with CKD, particularly in those with ESKD undergoing dialysis therapy, are susceptible to infection due in part to altered immune responses, multiple comorbidities, use of immunosuppressants, and use of vascular access (Ishigami and Matsushita, 2019), and hence a number of studies have reported the existence of bacterial DNA in the blood and its association with inflammatory response in these patients (Navarro et al., 2007; Bossola et al., 2009; Kwan et al., 2013; Shi et al., 2014). Nevertheless, there is a paucity of data on the characteristics and roles of the circulating microbiota in patients with CKD and ESKD. In a recent pilot study comparing the composition of circulating microbiota between non-diabetic CKD patients without kidney replacement therapy and healthy individuals, patients with CKD (vs. healthy individuals) displayed a significant reduction in α diversity (Chao1 index) and had a significantly higher relative abundance of Enterobacteriaceae and Pseudomonadaceae families, Gammaproteobacteria class, and Proteobacteria phylum in their buffy coat samples (Shah et al., 2019).

**Figure 1:**
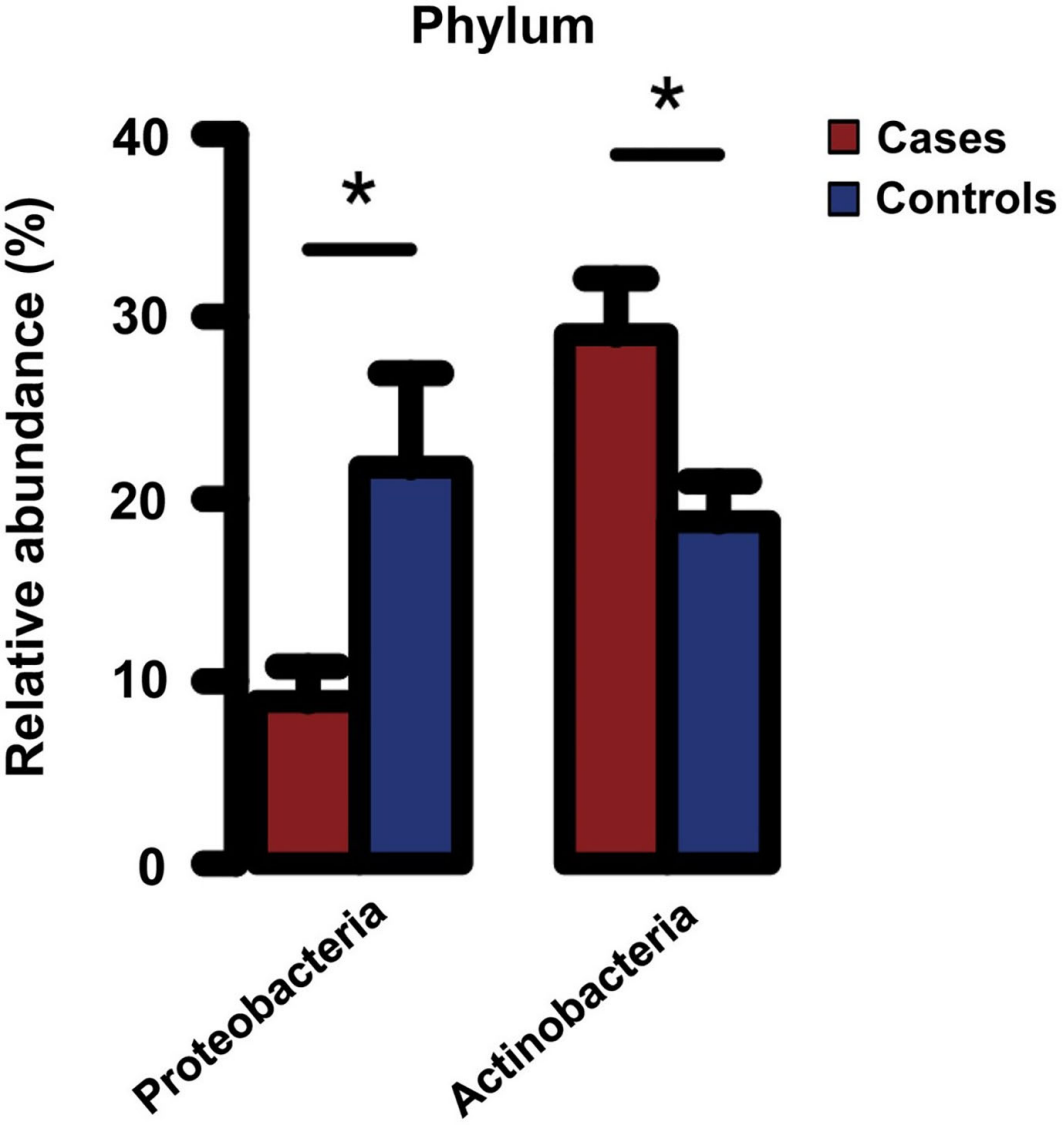
Relative abundance of Proteobacteria and Actinobacteria phyla in serum of hemodialysis patients with (cases) and without (controls) a fatal cardiovascular event. *P < 0.05. Error bars indicate the standard error of the mean (SEM). Reprinted with permission from Sumida et al. (2021b).

More recently, using serum samples of 34 hemodialysis patients enrolled in a pilot case-control study (17 cases with a fatal cardiovascular event and 17 matched controls without such an event during a median follow-up of 2.0 years), the researchers performed a comprehensive taxonomic profile of the circulating microbiota by 16S or ITS rRNA sequencing (for bacteria, archaea, and fungi) and compared the composition of circulating microbiota between cases and controls (Sumida et al., 2021b). They found that patients who died of a cardiovascular event (i.e., cases) had significantly less Proteobacteria and greater Actinobacteria phyla compared with those who remained alive (i.e., controls) (**Figure 1**), although the levels of 16S rRNA and bacterial α diversity were similar between groups (Sumida et al., 2021b). Furthermore, the proportion of Proteobacteria and Actinobacteria phyla were significantly correlated with blood levels of nuclear factor erythroid 2−related factor 2 (Nrf2), a master regulator of antioxidative responses, and were marginally associated with a greater risk of cardiovascular mortality, independently of age, sex, race, dialysis vintage, and type of vascular access (Sumida et al., 2021b). These data may suggest that circulating levels of Nrf2 play a key role in the risk of premature cardiovascular mortality associated with the circulating microbiota in patients with ESKD. Of note, this study utilized serum samples to assess the circulating microbiota, in spite of the perceived concern regarding the ability to detect microbial DNA in the cell-free fraction of blood. In most previous studies, the circulating microbiota was assessed using leukocyte-containing blood fractions (Navarro et al., 2007; Paisse et al., 2016; Shah et al., 2019), the nature of which may differ from that of the circulating microbiota identified in the cell-free blood fraction. More specifically, compared with circulating microbial signatures that can be identified predominantly in the buffy coat (rich in white blood cells and platelets) presumably as a result of the microbial entrapment by white blood cells, the circulating “cell-free” microbiota located in plasma or serum fraction may exert its potential pathophysiological effects on immune cells (through their receptors [e.g., TLR-9]) and on cardiac myocytes in a more direct manner, which in turn suggests the potential of the circulating cell-free microbiota as a more clinically applicable, non-invasive diagnostic/prognostic biomarker compared with the circulating microbiota identified from other leukocyte-containing blood fractions (Sumida et al., 2021b). The observed lower proportion of Proteobacteria phylum among cardiovascular cases (vs. controls) in this study may appear contradictory to the findings of other studies. Possible explanations include differences in blood fractions used to assess the circulating microbial composition, differences in the studied patient populations, with hemodialysis patients suffering from higher comorbidity burden, and/or differences in the relative abundance of other microbial taxa (e.g., high *Staphylococcus* genus which belongs to the phylum Bacillota) in cardiovascular cases in this study (Sumida et al., 2021b). Uncovering the cause(s) for these differences deserves further investigation. Nonetheless, a prior study that reported lower proportion of Proteobacteria phylum in subgingival plaque to be associated with higher systemic inflammation may support the finding from hemodialysis patients, suggesting a potential influence of oral microbiota on the circulating microbiota in these patients (Demmer et al., 2017).

## Future Directions and Challenges

It has now become evident that highly diverse microbial communities exist in the systemic circulation of various populations and that both quantitative and compositional changes in the circulating microbiota may contribute to the development and progression of cardiometabolic disease. However, many important questions remain unanswered regarding the nature of circulating microbiota, including the sources, pathophysiological roles, localization across different blood fractions, and distinction from potential contamination, all of which need to be clarified in future in-depth basic, clinical and population-based research. In an ongoing quest to improve outcomes of patients with cardiometabolic disease, perhaps the time has come to go beyond the “gut feeling” and rigorously incorporate the potential pathophysiological insights gained from the circulating microbiota towards the development of novel biomarkers for diagnosis and prognosis, especially in personalized therapeutic approaches to premature morbidity and mortality in cardiometabolic disease where patterns are emerging.

## Author Contributions

KS and JP: conception and study design and drafting the manuscript. ZH, C-YC, TM, AB, RD, SD, and CK: revising the manuscript critically for important intellectual content, and approval of the version of the manuscript to be published. All authors contributed to the article and approved the submitted version.

## Funding

This work was supported by the National Institute of Diabetes and Digestive and Kidney Disease (NIDDK) of the National Institutes of Health (NIH) under award number R01DK125586 to KS. JP is funded by NIH under award numbers R01CA253329 and R21AI163503. AB is funded by NIH under award number of R01DK117183. RD is funded by NIDDK under award number R01DK102932.

## Conflict of Interest

The authors declare that the research was conducted in the absence of any commercial or financial relationships that could be construed as a potential conflict of interest.

## Publisher’s Note

All claims expressed in this article are solely those of the authors and do not necessarily represent those of their affiliated organizations, or those of the publisher, the editors and the reviewers. Any product that may be evaluated in this article, or claim that may be made by its manufacturer, is not guaranteed or endorsed by the publisher.
